# Probability-based adaptive capacity rental strategy on shared platform with unknown demand distribution

**DOI:** 10.1371/journal.pone.0322837

**Published:** 2025-05-23

**Authors:** Yu Gong, Hui Yu

**Affiliations:** School of Economics and Business Administration, Chongqing University, Chongqing, PR China; Shandong University of Science and Technology, CHINA

## Abstract

Capacity sharing presents a transformative strategy in manufacturing, driven by the increasing demand for flexibility and efficiency in a highly uncertain market. Shared platforms play a crucial role in facilitating this transformation by offering a variety of scenarios that enable enterprises to make adaptable decisions. This paper develops a capacity sharing model on a shared platform, addressing two scenarios—standardized and differentiated scenarios—the latter incorporating cost discounts. We propose a probability-based adaptive rental strategy (PAS) in the absence of demand distributions. This strategy depicts human psychology and behavior through three steps: designing options, calculating probabilities, and establishing schemes. It differs from direct optimization of decisions by adaptively addressing stochastic problems through options and probabilities. Experiments demonstrate that PAS can balance flexibility and stability across diverse environments, including Poisson, Normal, multimodal, heavy-tailed distributions, and real-world datasets. Furthermore, it achieves near-optimal average profit performance, with improvements attainable through option adjustments.

## 1. Introduction

Shared manufacturing has become a pivotal force in the global industrial landscape, representing a substantial shift in practices. It is steering industries toward sustainable growth, acting as a driver of technological innovation. The World Manufacturing Report highlights the rapid adoption of shared manufacturing models, particularly as part of new business frameworks designed to optimize production capacities and address global supply chain challenges [1].

Capacity sharing is integral to the concept of shared manufacturing, strengthening resource utilization, market adaptability, and collaboration across the supply chain. It is primarily enabled by platforms or mechanisms that leverage technologies like cloud computing and the Internet of Things [2], supporting the sharing of production capabilities and resources (e.g., equipment, production lines, warehousing). Statista [3] projects that the global sharing economy will reach $335 billion by 2025, fueled by significant growth in recent years. This trend is also evident in the manufacturing sector, where leading solution providers have started to share their facilities and advanced operations to address companies’ outsourcing requirements [4].

Capacity sharing serves as a pivotal driver in the upgrading practices of global manufacturing. Several enterprises and platforms promote these practices. For instance, Xometry (https://www.xometry.com) is a prominent shared manufacturing platform that enables enterprises to quickly identify available production resources. Other platforms, like Flex (https://www.flex.com) and Protolabs (https://www.protolabs.com), offer equipment for shared resource use. Europe’s Industry 4.0 [5] initiative facilitates small and medium-sized enterprises (SMEs) with access to advanced manufacturing equipment through shared platforms, allowing them to produce high-quality goods comparable to those of large corporations while significantly reducing costs and boosting competitiveness. In China, Alibaba’s Tao Factory (https://tgc.tmall.com) platform connects micro and small enterprises, allowing them to share information about their resources, including equipment types, idle capacity, and available time. Similarly, JD Manufacturing (https://www.jd.com) and Pin Factory from PDD (https://www.pinduoduo.com) empower manufacturers to collaborate with complementary businesses to fulfill orders when their own capacity is insufficient. In 2022, Shared Factory (https://www.sharedfactory.com), China’s largest online on-demand production platform, was established to unite various manufacturing enterprises, offering a wide array of shared services such as computer numerical control (CNC) machining, metal parts production, and 3D printing.

Shared platforms facilitate capacity sharing among enterprises (Choudary et al. [6]), while various digital technologies significantly accelerate collaboration within global supply chains [7]. Researchers and practitioners propose different classification methods to help participants select and build suitable platforms. We draw on the classification from [2], which categorizes these platforms into four types based on their initiators, structures, and functions: intermediary platforms, co-creation platforms, service-oriented platforms, and collaborative platforms. (Table 1). Intermediary platforms are the most common and offer high flexibility and efficient resource allocation. Co-creation platforms, also initiated by large firms, emphasize professional expertise and innovation. Service-oriented platforms, led by industrial technology companies, help businesses achieve digital transformation. Collaborative platforms, formed by SMEs, focus on reducing costs through shared resources and collective efforts.

**Table 1:** Four types of platforms for capacity sharing.

Type	Established	Service Characteristic	Examples
Intermediary Platforms	Internet entrepreneurs	Provide matchmaking services to bridge the gap between suppliers and users; do not own equipment or production facilities	Tao Factory Xometry
Co-creation Platforms	Industry leaders	Offer comprehensive solutions, integrating online platforms with incubation services	HCH BEACON
Service-oriented Platforms	Manufacturing enterprises with complete capabilities	Supply equipment, production lines, and other resources, and also provide software, intelligent control, and cloud services	iSESOL Fii Cloud
Collaborative Platforms	Multiple SMEs from the same region or industry	Share equipment, production lines, and plant buildings, as well as human resources, to improve efficiency and reduce costs	ShengYiBang Shared Factory

The platforms expand the methods for capacity sharing among enterprises, with the rental of production resources as a common practice. They provide or match for the rental of equipment, production lines, and other resources. Unlike traditional rental arrangements, capacity sharing through platforms is demand-driven and does not require ownership of production resources [8,9]. Different platforms adopt various pricing models, which primarily include rental fees, service fees, membership fees, and transaction fees. The rental fee, payable to suppliers, is based on the hours of use or actual utilization, allowing enterprises to align decisions with operational needs.

This paper examines two scenarios: the standardized and differentiated. In the standardized scenario, platforms offer shared production resources that adhere to uniform specifications, resulting in consistent production costs. For instance, intermediary platforms consolidate manufacturers with comparable resources and match them with enterprises in need, thereby ensuring consistency in production processes and quality, like Maschinenring (https://www.maschinenring.de). In the differentiated scenario, platforms offer a variety of production resources with costs that vary by type. Generally, smaller-scale production resources incur higher costs than larger-scale options. Certain platforms provide discounts based on production volume to encourage larger orders and foster collaboration. Intermediary platforms may pair smaller orders with small or semi-automated equipment, while larger orders are matched with fully automated, high-efficiency machinery, as seen with Xometry and Tao Factory.

The trend of capacity sharing highlights the rising need for enterprises to adapt to market uncertainties. Various platforms provide convenience in capacity sharing; however, enterprises are confronted with rapidly changing data, complicating their ability to obtain comprehensive information. Enterprise face decision-making challenges due to incomplete information and better interaction with the platform. Our analysis addresses the following questions: (1) How can a strategy be modeled on shared platforms under unknown demand distributions? (2) What are the characteristics and performance of this strategy in a standardized scenario? (3) What effect does the strategy have when cost discounts are present in a differentiated scenario?

To address these problems, this paper establishes a model in which enterprises engage in capacity sharing through platforms. The platforms offer two types of rental scenarios: the first is a standardized scenario, in which enterprises rent identical equipment at a constant unit cost; the second is a differentiated scenario, where orders exceeding a specific threshold can access larger equipment and benefit from cost discounts. We introduce a probability-based adaptive strategy (PAS) to assist enterprises in making rental decisions under unknown demand distributions, adaptable to both scenarios. This strategy mimics human decision-making behavior by transforming direct rental decisions into leveraging probabilities of options (dual reference points) to adapt problems. Through numerical experiments, this study analyzes the characteristics and performance of PAS across different distributions and the real-world dataset, comparing it to benchmarks.

The structure is organized as follows. Section 2 offers a comprehensive review of the relevant literature. Section 3 delineates the problem, develops models for two scenarios, and provides an explanation of PAS. In Section 4, experiments are conducted in two scenarios with Poisson and normal distributions. Section 5 introduces more volatile multimodal and heavy-tailed distributions, incorporating the real-world dataset to compare and analyze the characteristics and performance of the PAS from multiple perspectives. Section 6 concludes the paper and outlines future research.

## 2. Related work

### 2.1 Impacts and strategies for capacity sharing platforms

The first literature stream relevant to this research considers strategies for capacity sharing platforms, highlighting the interactions among suppliers, platforms, and users. One type of research focuses on the impact of platforms on participants and the roles they play. Li et al. [10], Ma and Xie [11], and Zhao et al. [12] contribute to this discourse by categorizing and comparing the effects of having a platform versus not having one on the profits for both sides. For instance, Ma and Xie [10] investigate the benefits of capacity sharing for suppliers and users, as well as how different matching success rates influence suppliers’ decisions regarding spontaneous matching through the platform. Zhao et al. [12] explore channel selection and pricing strategies for manufacturers aiming for sustainable operations, focusing on charging modes, access requirements, and commission rates across various stages of platform development.

Another type of research examines how various factors affect decisions related to capacity sharing within platforms [13–17]. Wu and Liu [13] access how cooperative advertising affects pricing and profits in a shared manufacturing platform, presenting models of traditional cooperation, cost-sharing contracts, revenue-sharing contracts, and bilateral cost-sharing contracts. Han and Yao [15] analyze the preferences of capacity suppliers, users, and platforms regarding different trading strategies from the perspectives of profitability and stability. The study reveals that platforms prioritize stability over profitability, even though the latter may yield higher expected profits. Li et al. [16] consider whether a platform should establish quality admission standards and how to set these thresholds if implemented.

Research on strategies in capacity-sharing platforms often emphasizes pricing strategies and capacity allocation decisions. Li et al. [16] specifically address how to determine platform service prices and capacity rental prices under varying quality admission scenarios, along with the product sales pricing for both suppliers and users. He et al. [17] aim to understand how cross-network externalities and supply risks in a bilateral market shape pricing decisions for capacity-sharing platforms and the adoption of blockchain technology. Li et al. [18], Xie et al. [19], and Zhang et al. [20] present innovative allocation strategies for capacity sharing. Li et al. [18] enhance a novel energy-sharing mechanism, while Xie et al. [19] introduce an optimal or near-optimal matching algorithm that employs a two-dimensional crossover and an order-first mutation using genetic algorithms (GA), demonstrating the effectiveness of both algorithms for various schemes. Zhang et al. [20] propose the capacity matching problem with time windows and order splitting, designing a two-stage heuristic algorithm to solve the model. Other papers simultaneously consider pricing and capacity quantity decisions within their models ([10–13,15,16,21]).

Existing literature thoroughly examines the impact of shared platforms on enterprises and the various factors influencing pricing and capacity decisions (Li et al. [14] also address wages, customer surplus, and labor welfare). This study focuses on specific renting scenarios provided by the platform, highlighting how enterprises develop flexible and adaptive rental strategies that align with stochastic demands. From a methodological perspective, most studies mainly employ game theory, with a few studies incorporating heuristic algorithms [19,20]. While this approach emphasizes interactions among stakeholders, it inadequately captures the complexities of market dynamics due to linear demand expressions. This paper characterizes the incomplete nature of demand information in its strategy, thereby deepening the understanding of operational decisions in practice.

### 2.2 Rental and capacity strategies in capacity sharing

The first part of this literature stream examines research related to rental issues. Renting is a prevalent approach within capacity sharing, particularly emphasized in studies across the energy [22,23], electricity [24,25], 3D printing [26], and transportation [27,28] sectors. These studies directly analyze the benefits of rental strategies, along with decisions regarding pricing and amounts of capacity. Li et al. [22] introduce a novel business model for a Virtual Storage Rental Service (VSRS), where users aim to minimize electricity costs by renting virtual capacity and optimizing operations. Klampfer and Chowdhury [24] present a method for addressing bottlenecks through the dynamic sharing or renting of service capacity, minimizing the number of rented lines. Sun et al. [26] explore the role of capacity pricing in matching supply and demand, emphasizing the optimal pricing strategy for the platform and examining how usage levels and printer heterogeneity influence consumer choices between in-house printing and outsourcing. Jiao and Ramezani [27] tackle fragmentation in the multi-platform ride-hailing market by proposing a dynamic cooperation mechanism that allows platforms to refer and lease passengers.

The second part adopts a broader perspective on capacity decisions, moving beyond the direct expression of rental capacity. These studies emphasize the use of sharing platforms or mechanisms to determine and allocate capacity more rationally and effectively. Some papers discuss how to allocate shared capacity among multiple enterprises, particularly competitive players, to optimize resource allocation and maximize profits [29–32]. For instance, Chen et al. [29] delve into the capacity sharing between two enterprises within Cournot competition and develop an optimal revenue-sharing contract. Their findings suggest that while capacity sharing can increase corporate profits, it does not fully alleviate the negative impacts on consumer surplus and social welfare. Other papers involve in how various entities determine their capacity decisions; when a platform is involved, researchers may also address its role in capacity matching [21,33–35]. Zhao et al. [33] concerned with the decision-making behaviors and profitability of suppliers, manufacturers, and platform operators under fixed transaction fee and quality-based transaction fee strategies. Wei and Zhang [34] develop a model consisting of two symmetric enterprises and compare their optimal capacity strategies under both capacity sharing and non-sharing scenarios. Notably, they find that delayed flexibility and capacity sharing can be either complementary or substitutive. Aloui and Jebsi [21] discuss how platforms balance cross-externalities and congestion costs among different groups during capacity allocation.

Current research targets capacity rental decisions in sectors like energy and electricity, while broader studies tend to frame capacity decisions in terms of sharing. Likewise, most of these studies emphasize game theory and interactions between two or more enterprises, often neglecting the demand environment. In contrast, our paper investigates how users determine the amount of rental capacity in a stochastic environment with unknown demand distributions using shared platforms.

### 2.3 Stochastic decision-making under unknown distributions

Stochastic decision-making under unknown distributions presents significant challenges, particularly when accurate data distributions are lacking, rendering traditional models ineffective. Researchers propose various approaches to address this issue, including Bayesian theory, regret theory, fuzzy decision-making, robust optimization, and artificial intelligence. Regret theory, introduced by Leonard J. Savage in 1951, posits that decision-makers evaluate their choices by comparing actual outcomes with expected results, which may lead to feelings of regret [36]. The minimax regret method identifies the minimum of the maximum regret across all possible outcomes, encouraging decision-makers to select options with the least regret value. In recent years, regret theory, particularly the minimax regret approach, has found widespread application in business operations, encompassing areas such as procurement, production, and inventory control [37–40]. These approaches facilitate firms to develop robust strategies in information-limited environments, thereby reducing operational risks and strengthening market competitiveness.

In tackling stochastic decision problems with unknown distributions, another set of methods emphasizes the cognitive, emotional, and psychological limitations faced by human decision-makers. These methods recognize that decision-makers are not perfectly rational agents; instead, they encounter constraints in information processing and decision-making. A prominent theory in this context is Simon’s Bounded Rationality Theory [41], which posits that decision-makers often lack essential resources, such as time, information, and cognitive capacity. Furthermore, they are influenced by emotional and psychological biases, leading them to prefer satisficing strategies over optimal ones. Simon also outlines the fundamental steps of decision-making, including information gathering, options generation, and selecting the best course of action based on analysis and preferences.

This paper proposes a new decision-making framework to address uncertainty. This strategy leverages data-driven options and stochastic probabilities to simulate human psychology and behavior. While regret theory and bounded rationality provide valuable theoretical references, this strategy distinguishes itself by relaxing the pursuit of optimal solutions and integrating mathematical modeling with data-driven insights. It prioritizes adaptability, allowing for flexible and stable adjustments in environments.

## 3. Problem description and model building

This paper analyzes capacity sharing models involving capacity suppliers, a shared platform, and a capacity user. In this model, suppliers offer equipment through the shared platform, while users rent capacity to meet market demand. The platform stipulates charges based on the amount of rented capacity, billing per unit of output. We develop two scenarios: the first is standardized, where suppliers offer a fixed cost per unit of production using identical equipment. The second is differentiated, in which suppliers provide both high-capacity and low-capacity equipment, with the latter being more efficient and having a lower unit cost. Users can benefit from cost discounts upon reaching a specified threshold when using the high-capacity equipment.

### 3.1 The standardized scenario

**Fig 1:**
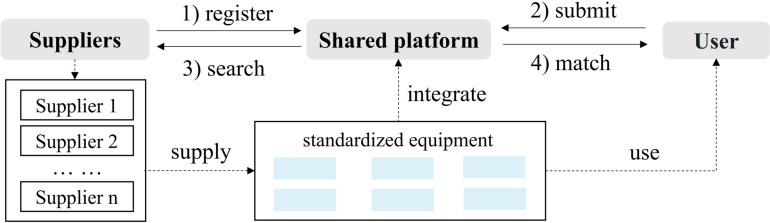
Basic framework for the standardized scenario.

The first scenario (Fig 1) involves identical types of equipment (Type I), with a unit cost of *c*_*s*_. The user faces uncertain market demand *d* each period, with a unit sales price of *p*, a salvage value of *s* for unsold goods, and a goodwill loss of *l*. for unmet demand. In each period, the user needs to decide on the amount of capacity to rent. A benchmark is first established for comparative analysis.

The platform Maschinenring (MR) aggregates a multitude of suppliers, enabling it to match users with several pieces of equipment that share consistent specifications. Suppliers list their available equipment with details on specifications and rental terms. Users search for equipment that meets their production needs and can reserve it through the platform. Once booked, users gain access to the equipment, which is monitored for performance during use. After the rental period, equipment is returned to the supplier, and users have the opportunity to provide feedback on their experience. MR employs a pay-per-use model, charging users based on the actual hours or the amount of output. It maintains standardized rates for similar types of equipment, ensuring predictable costs for users.

**The benchmark:** A benchmark model under complete information is established, where the distribution function and probability density function of demand are known as *F* and *f*. The variable ^*q*^_*ST*_ denotes the amount of capacity rented by enterprise, and the objective function is:


$$\pi_{ST}(q_{ST})=(p-s+l)E_{F}[\min(d,q_{ST})]-(c_{s}-s)q_{ST}-lE_{F}(d)$$ (1)

In this case, the optimal solution is $q_{ST}^{*}=F^{-1}(\frac{p+l-c_{s}}{p-s+l})$.

### 3.2 The differentiated scenario

**Fig 2:**
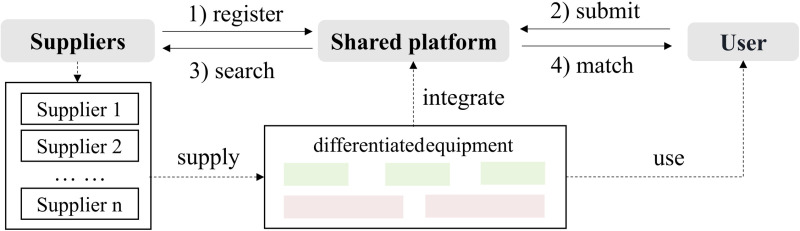
Basic framework for the differentiated scenario.

The second scenario (Fig 2) involves renting differentiated equipment, with large equipment (Type II) and small equipment (Type III). The unit costs of the two types of equipment are ^*c*^_*l*_ and ^*c*^_*h*_ (^*c*^_*l *_>* *^*c*^_*h*_), respectively. Once the amount of rented capacity meets a threshold ^*q*^_*TH*_, a cost discount is applied, allowing the use of Type II; otherwise, only Type III can be used. Other parameters are the same as above.

The platform Xometry offers equipment of various scales and capacities, enabling users to select machines that meet their specific requirements. For high-volume orders, Xometry typically provides discounts on the cost per unit. Large-scale production helps lower the per-unit cost, as fixed expenses—such as setup and maintenance of large equipment—can be distributed over a greater output. On the platform, suppliers register their available equipment, ensuring compliance with the platform’s requirements. When users submit their production needs, Xometry matches the orders with suitable suppliers based on location and equipment type. Users can choose from different equipment options and place orders through the platform while tracking their order status. After delivery, users can provide feedback.

**The benchmark:** In the differentiated scenario, achieving the threshold ^*q*^_*TH*_, will provide a cost discount. The variable ^*q*^_*DI*_, indicates the amount of capacity rented by enterprise, and the objective function is as follows:


$$\begin{array}{c} \pi_{DI}(q_{DI})=\left\{ \begin{array}{c} (p-s+l)E_{F}[\min(d,q_{DI})]-(c_{l}-s)q_{DI}-lE_{F}(d),q_{DI}\geq q_{TH}\\ (p-s+l)E_{F}[\min(d,q_{DI})]-(c_{h}-s)q_{DI}-lE_{F}(d),0<q_{DI}<q_{TH} \end{array}\right. \end{array}$$ (2)

**Proposition 1.** Let $q_{1}=F_{DI}^{-1}(\frac{p+l-c_{l}}{p-s+l})$ and $q_{2}=F_{DI}^{-1}(\frac{p+l-c_{h}}{p-s+l})$. In the differentiated scenario with complete information, the optimal solution satisfies:

(i). If $\left(p+l-c_{h})q_{2}-(p+l-c_{l})q_{1}\geq (p+l-s)[\int_{0}^{q_{2}}F(x)dx-\int_{0}^{q_{1}}F(x)dx\right]$ and $q_{1}\geq q_{TH}\geq q_{2}$, or $\left(p+l-c_{h})q_{2}-(p+l-c_{l})q_{TH}\geq (p+l-s)[\int_{0}^{q_{2}}F(x)dx-\int_{0}^{q_{TH}}F(x)dx\right]$ and $q_{TH}\geq q_{1}\geq q_{2}$, then

$q_{DI}^{*}=q_{2}=F_{DI}^{-1}(\frac{p+l-c_{h}}{p-s+l})$

(ii). If $\left(p+l-c_{h})q_{2}-(p+l-c_{l})q_{1}\leq (p+l-s)[\int_{0}^{q_{2}}F(x)dx-\int_{0}^{q_{1}}F(x)dx\right]$ and $q_{1}\geq q_{TH}\geq q_{2}$, or $q_{1}\geq q_{2}\geq q_{TH}$, then

$q_{DI}^{*}=q_{1}=F_{DI}^{-1}(\frac{p+l-c_{l}}{p-s+l})$

(iii). If $\left(p+l-c_{h})q_{2}-(p+l-c_{l})q_{1}\leq (p+l-s)[\int_{0}^{q_{2}}F(x)dx-\int_{0}^{q_{TH}}F(x)dx\right]$ and $q_{TH}\geq q_{1}\geq q_{2}$, then

$q_{DI}^{*}=q_{TH}$

### 3.3 The probabilistic-based and adaptive strategy

Although optimal decisions based on probability distributions strengthen planning and stability, their effective implementation depends heavily on precise demand predictions, which are often difficult to achieve. Complete information about demand distribution is often difficult to obtain, typically leaving only partial historical data available. Moreover, it is well-established that human decision-making processes are influenced by psychological factors, and the pursuit of optimality is not absolute.

Perakis and Roels [39] proposed a minimax regret approach under unknown distributions, which not only captures the decision-maker’s regret aversion but also utilizes partial information. Simon introduced the concept of bounded rationality, acknowledging the various limitations humans face in decision-making and their tendency to seek a ‘good enough’ solution. To formalize this idea, Simon outlined a four-step process to better model human decision-making behavior.

This paper proposes a novel approach, termed the probability-based adaptive strategy (PAS), for stochastic problems under partial information, integrating insights from regret theory and bounded rationality. From an intrinsic logic perspective, PAS simulates human psychological and behavioral factors to make adaptive decisions. Rather than directly determining the variables, it reformulates the problem as a combination of stochastic probabilities and options. The options (dual reference points) are data-driven and integrate human knowledge, while probabilities are calculated using minimax regret to adapt to environments. In terms of external characteristics, PAS balances flexibility and stability. Flexibility, driven by stochastic probabilities, enables adjustments based on environmental changes. Stability, ensured by dual reference points, anchors the variation range within two options, enhancing the strategy’s controllability.

**Fig 3:**
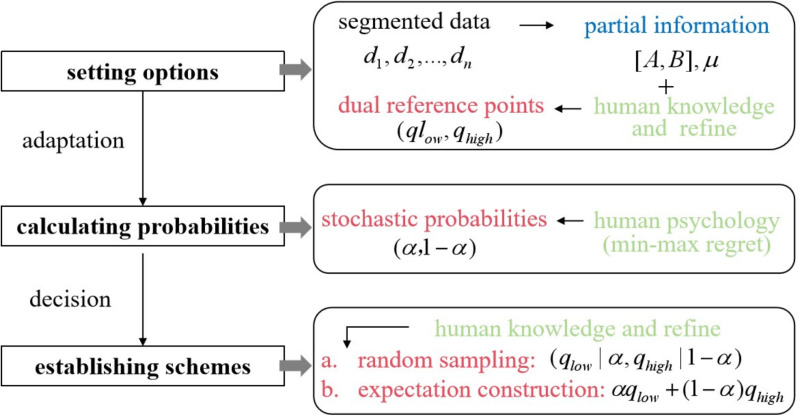
The logical structure of PAS.

Specifically, PAS structures decision-making into three steps: (1) designing options, (2) calculating probabilities, and (3) establishing schemes, thereby emulating human decision psychology and behavior (Fig 3). The first and third steps highlight the role of available data and refined human knowledge. In the second step, PAS emphasizes ‘addressing uncertainty with uncertainty,’ where two options serve as dual reference points, and the selection and adjustment occur adaptively through probability.

(1) **Designing options**

This paper presents a data-driven and human-refined approach to the design of options. When decision-makers have access to partial data streams, they often use statistical analysis to support decision-making. Although such information is limited, it remains valuable in providing actionable insights under resource constraints. The procedure is detailed below.

Two options for the amount of capacity are assumed, denoted as ^*q*^_*low*_ and ^*q*^_*high*_ (^*q*^_*low* _<*q*_*high*_). First, samples are drawn from historical demand data, and the bootstrap is employed to statistically estimate the mean and standard deviation. They will assist in preliminarily estimating the overall level of demand, enabling the choice of appropriate equipment. Next, a linear function $\left(q_{low},q_{high})=(m_{1}\hat{\mu }-n_{1}\hat{\sigma },m_{2}\hat{\mu }+n_{2}\hat{\sigma }\right)$ will be constructed based on the estimated features to determine the options, where *m*_*1*_*, m*_*2*_ and *n*_*1*_*, n*_*2*_ are adjustment coefficients used to accommodate different environments. Meanwhile, if suitable equipment is matched, options can be designed by variations centered around the capacities of different equipment, like $\left(q_{low},q_{high})=(m_{1}q_{s}-n_{1}\hat{\sigma },m_{2}q_{s}+n_{2}\hat{\sigma }\right)$ or $\left(q_{low},q_{high})=(m_{1}q_{l}\pm n_{1}\hat{\sigma },m_{2}q_{h}\pm n_{2}\hat{\sigma }\right)$. It is vital to highlight that experiments will illustrate that the aim of this method is not to derive precise values for options, but rather to offer an adaptive framework. In other words, diverse combinations of options can be effective.

(2) **Calculating probabilities**

Stochastic probability lies at the core of decision-making, facilitating adaptive adjustments to environmental dynamics by learning characteristic information, which guides the selection between dual reference points. The research by Perakis and Roels [39] provides valuable insights, which introduce the concept of minimax regret and offer methodologies for making decisions under partial information.

We construct the objective function for minimax regret and compute the probability *α*. Following the foundational proposition 1(a)(b) by Perakis and Roels [39], the regret value is defined as $\rho =\underset{\beta \in [0,1]}{\max}\;\left\{\prod_{F}[\beta ]\right\}-\prod_{F}[\alpha ]$, while the maximum regret is $\underset{F\in 𝒟}{\max}\;\underset{\beta \in [0,1]}{\max}\;\left\{\prod_{F}[\beta ]\right\}-\prod_{F}[\alpha ]$. Where Δ denotes the convex set of distributions *F*, characterized by specific moments and shapes. Since only characteristic information about demand can be inferred from the data stream, this paper employs the upper and lower bounds *[A, B]*, along with the mea*n μ.* It specifies that *μ*<^*q*^_*low* _<^*q*^_*high*_, with the variable ^*q*^_*DI*_ representing the amount of rented capacity, $q_{DI}=\left\{ \begin{array}{c} qlow,\alpha \\ q_{high},1-\alpha \end{array}\right.$. The profit and the minimax regret functions are given by Equation (3)–(5):


$$\begin{array}{c} \pi_{DI}(q_{low},q_{high}|\alpha )=\left\{ \begin{array}{c} (p-s+l)E_{F}[\min(d,q_{low})]-(c_{h}-s)q_{low}-lE_{F}(d)=\pi_{1}(q_{low}),\alpha \\ (p-s+l)E_{F}[\min(d,q_{high})]-(c_{l}-s)q_{high}-lE_{F}(d)=\pi_{2}(q_{high}),1-\alpha \end{array}\right. \end{array}$$ (3)

$$E[\pi_{DI}(q_{low},q_{high}|\alpha )]=\alpha E[\pi_{1}(q_{low})]+(1-\alpha )E[\pi_{2}(q_{high})]$$ (4)

$$\rho^{*}(\alpha )=\underset{\alpha \in [0,1]}{\min}\;\left\{\rho (\alpha )\right\}=\underset{\alpha \in [0,1]}{\min}\;\underset{F\in 𝒟}{\max}\;\underset{\beta \in [0,1]}{\max}\;\left\{\pi_{DI}[\beta ]\right\}-\pi_{DI}[\alpha ]$$ (5)

Solving Equation (5) requires hierarchical decomposition and we restructure it into $\underset{\alpha \in [0,1]}{\min}\;\underset{\beta \in [0,1]}{\max}\;\left\{\underset{F\in 𝒟}{\max}\;\left\{\pi_{DI}[\beta ]-\pi_{DI}[\alpha ]\right\}\right\}$. The innermost problem is transformed into a common form using duality (Equation (6)). The two outer problems are then solved sequentially. A more detailed derivation process will be presented in the appendix.


$$\begin{array}{cc} & \underset{a_{0},...,a_{n}}{\min}\;\begin{array}{cc} & \sum {\mathrm{\backslash nolimits}}_{i=0}^{n}a_{i}M_{i} \end{array}\\ & s.t.\begin{array}{cc} & \sum {\mathrm{\backslash nolimits}}_{i=0}^{n}a_{i}x^{i}\geq (\beta -\alpha ) \end{array}(p-s+l)[\min(q_{low},x)-\min(q_{high},x)] \end{array}$$ (6)

Let M_i_ be the known partial information (usually the moment). According to the complementary slackness conditions, achieving the optimal in Equation (6) requires that $\sum {\mathrm{\backslash nolimits}}_{i=0}^{n}a_{i}x^{i}-(\beta -\alpha )(p-s+l)[\min(q_{low},x)-\min(q_{high},x)]=0$, which finds the intersection point of $\sum {\mathrm{\backslash nolimits}}_{i=0}^{n}a_{i}x^{i}$ and $\left(\beta -\alpha )(p-s+l)[\min(q_{low},x)-\min(q_{high},x)\right]$. We present and analyze the results in Proposition 2 and Corollaries 1 and 2.

In PAS, *q*_*DI*_ serves as the dual reference point. It enhances clarity by providing baselines and reduces uncertainty by anchoring choices within well-defined lower and upper bounds. The probability α allows for a more nuanced understanding of potential outcomes. By incorporating new information over time, it supports flexible and adaptive adjustments to decision-making. This approach enhances adaptability and emphasizes the value of data-driven methods.

**Proposition 2.** If the demand distribution is nonnegative, with support *[A, B]* and mean *μ*, the minimax regret of α^*^ satisfies:

(i) if $(c_{l}-s)q_{high}-(c_{h}-s)q_{low}>\frac{\left(\mu -A)(p-s+l)(q_{high}-q_{low}\right)}{q_{high}-A}$, then $\alpha^{*}=1$.
(ii) if $0\leq (c_{l}-s)q_{high}-(c_{h}-s)q_{low}\leq \frac{\left(\mu -A)(p-s+l)(q_{high}-q_{low}\right)}{q_{high}-A}$, then

$\alpha^{*}=\frac{\left[(c_{h}-s)q_{low}-(c_{l}-s)q_{high}](q_{high}-A\right)}{\left(\mu -A)(p-s+l)(q_{low}-q_{high}\right)}$

(iii) if $(c_{l}-s)q_{high}-(c_{h}-s)q_{low}<0$, then $\alpha^{*}=0$.

From Proposition 2, it is observed that the upper bound does not affect the calculation of *α*, while the lower bound and the mean do. If the net cost of ^*q*^_*high*_ is lower than ^*q*^_*low*_, then ^*q*^_*high*_ will be chosen directly; if the difference between the two exceeds the $\frac{\left(\mu -A)(p-s+l)(q_{high}-q_{low}\right)}{q_{high}-A}$, ^*q*^_*low*_ will be selected. For the condition (ii), the impact of different parameters on α^*^ can be identified based on Corollary 1.

**Corollary 1**. Let $0\leq (c_{l}-s)q_{high}-(c_{h}-s)q_{low}\leq \frac{\left(\mu -A)(p-s+l)(q_{high}-q_{low}\right)}{q_{high}-A}$, the α^*^ satisfies:

(i) α^*^ increase with ^*c*^_*l*_ and *A*.
(ii) α^*^ decrease with ^*c*^_*h*_, *p*, *l*, *μ*, and ^*q*^_*low*_.
(iii) if $\frac{q_{high}}{q_{low}}\leq \frac{p+l-c_{h}}{p+l-c_{l}}$, α^*^ increase with *s*, otherwise, it decreases.
(iv) if $(c_{h}-s)q_{low}-(c_{l}-s)q_{high}\geq \frac{\left(c_{l}-s)(q_{high}-A)(q_{low}-q_{high}\right)}{q_{low}-A}$, α^*^ increase with ^*q*^_*high*_, otherwise, it decreases.

Under the condition of ^*c*^_*h=*_^*c*^_*l=*_^*c*^_*s*_, the differentiated scenario can be converted into a standardized scenario (Corollary 2). At this point, the value of α^*^ is not related to ^*q*^_*low*_ and *B*.

**Corollary 2**. Let ^*c*^_*h=*_^*c*^_*l=*_^*c*^_*s*_. The α^*^ in Proposition 2 satisfies:

(i) if $c_{s}-s>\frac{\left(\mu -A)(p-s+l\right)}{q_{high}-A}$, then $\alpha^{*}=1$.
(ii) if $0<c_{s}-s\leq \frac{\left(\mu -A)(p-s+l\right)}{q_{high}-A}$, then $\alpha^{*}=\frac{\left(c_{s}-s)(q_{high}-A\right)}{\left(\mu -A)(p-s+l\right)}$.
(3) **Establishing schemes**

We introduce human refinement to assist in determining the final decision. When utilizing probabilities for option selection, three common schemes are employed: selecting the option with the highest probability, sampling based on probability, and combining probabilities with options to form expected values. This paper draws on the latter two schemes.

The first is the random sampling, which involves selecting ^*q*^_*low*_ and ^*q*^_*high*_ based on the probability α^*^. In this case, the decision is based on a two-point distribution, which further simplifies the decision-making process, allowing enterprises to manage stochastic demand with just two values. This also leads to a question: why not directly choose the option with the highest probability rather than random sampling? When the probability approaches 0.5, identifying the superior one becomes difficult. Random sampling offers greater reliability from a probabilistic perspective.

The second scheme is the expectation construction, where the decision is determined by *α*^*q*^_*low*_+(*1*-*α*)^*q*^_*high*_. This scheme thoroughly considers the impact of each option on decision-making. It allows for flexible numerical adjustments and fosters a more resilient strategy. From a long-term perspective, it is likely to generate more stable returns. This is particularly advantageous in dynamic environments. Furthermore, the use of expectation construction can increase clarity in decision-making grounded in a mathematical framework.

## 4. Experiments in normal and poisson distributions

This section evaluates the features and performance of PAS in stochastic demand environments through numerical experiments. Demand is simulated using Poisson and Normal distributions, which are widely applied in related research (Table 3). Each experiment runs for 200 periods. All experiments described in Sections 4 and 5 are conducted using Python, with the libraries NumPy, pandas, SciPy, and Matplotlib.

**Table 3:** Parameter settings in two scenarios.

d	p	l	s	$c_{s}$	$c_{l}$	$c_{h}$	$q_{low}$	$q_{high}$
P(10), $N(10,2^{2})$	5	2	1	3	/	/	7,8	13,12
$N(50,10^{2})$	10	2	1	/	8	6,4	40	60

### 4.1 Experiments in standardized scenario

A total of 20 sets of sample data, each containing 50 samples, are generated randomly. In the Poisson distribution, the estimated mean and standard deviation are 9.95 and 3.12, respectively, while in the Normal distribution, they are 9.987 and 2.056. The initial options under the two distributions are set as ^*q*^_*low*_ = 7, ^*q*^_*high*_ = 13 and ^*q*^_*low*_ = 8, ^*q*^_*high*_ = 12 (*m*_*1*_ = *m*_*2*_ = *n*_*1*_ = *n*_*2*_ = 1).

#### 4.1.1 Performance of fixed options.

**Fig 4:**
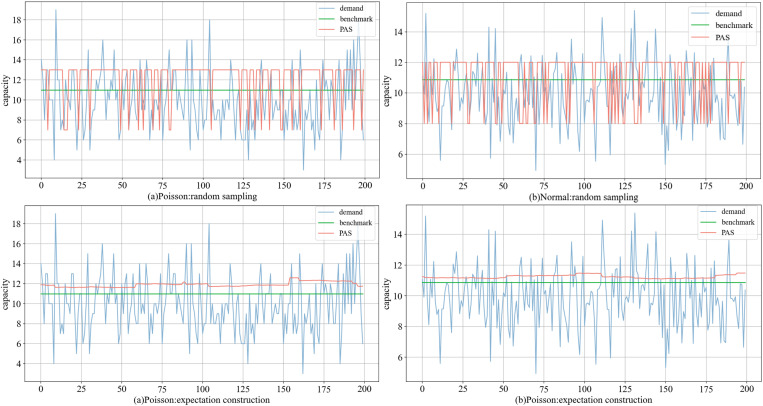
Comparison of decisions under two schemes in (a) the Poisson and (b) the Normal distribution.

Fig 4 (group 4) illustrates the comparison of decision-making between PAS and the benchmark. PAS can sense fluctuations in demand and adjusts its choices accordingly. On average, the amount of capacity rented by PAS are upper than those of the optimal solution in Poisson and Normal distributions. Furthermore, PAS infers future demand conditions based on historical data stream, which may introduce a phenomenon of delayed response. As indicated by Proposition 2 and Corollary 1, the loss of goodwill exceeds the salvage, leading PAS to favor renting more capacity (the probability α decreases). Whether using the Poisson (discrete) or the Normal distribution (continuous), the expectation construction scheme better adapts to demand changes and offers greater stability. The random sampling scheme relies on only two values, facilitating standardized and simplified decision-making.

Table 4 presents the average profit ($(1/Tsum{\mathrm{\backslash nolimits}}_{i=1}^{T}\pi_{t}$) of PAS and the benchmark over 10 simulations. PAS using the expected construction achieves an average profit closely aligned with the benchmark. For the Poisson distribution, the highest radio reaches 0.999, while for the Normal distribution, it is 0.998. Although random sampling yields slightly lower results, achieving 0.869 and 0.944, respectively, it still performs well. From the average, the two schemes show radio of 0.836 and 0.978 in the Poisson distribution, and 0.914 and 0.991 in the Normal distribution. Even in the worst-case conditions, PAS can achieve 75% of the optimal solutions.

**Table 4:** Average profits in Poisson and Normal distributions under two schemes.

P(10)	benchmark	random sampling		expectation construction	
	value	value	radio	value	radio
1	13.300	11.520	0.866	13.011	0.978
2	13.980	11.390	0.815	13.689	0.979
3	12.450	10.100	0.811	11.963	0.961
4	13.610	11.770	0.865	13.230	0.972
5	12.710	11.050	0.869	12.573	0.989
6	13.310	10.720	0.805	13.309	0.999
7	13.190	10.900	0.826	12.905	0.978
8	13.130	11.260	0.858	12.997	0.990
9	12.910	10.170	0.788	12.376	0.959
10	12.600	10.820	0.859	12.271	0.974
**Ave**	**13.119**	**10.970**	**0.836**	**12.832**	**0.978**
$N(10,2^{2})$	**value**	**value**	**radio**	**value**	**radio**
1	16.034	14.766	0.921	15.947	0.995
2	15.935	14.829	0.931	15.790	0.991
3	16.110	14.899	0.925	15.962	0.991
4	15.646	13.929	0.890	15.456	0.988
5	16.010	14.325	0.895	15.817	0.988
6	16.001	14.728	0.920	15.931	0.996
7	15.550	14.682	0.944	15.514	0.998
8	15.611	14.168	0.908	15.432	0.989
9	15.524	14.192	0.914	15.414	0.993
10	15.170	13.465	0.888	14.964	0.986
**Ave**	**15.759**	**14.398**	**0.914**	**15.623**	**0.991**

* Please note that all results reported in this paper are presented to three decimal places.

**Fig 5:**
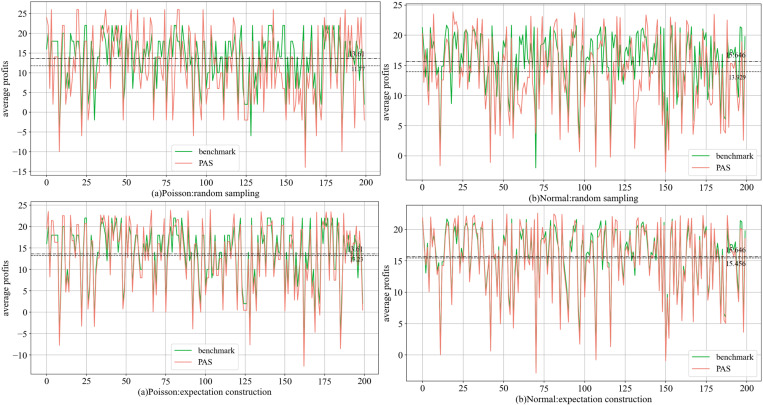
Comparison of average profits under two schemes in (a) the Poisson and (b) the Normal distribution.

Using the group 4 as an example (Fig 5), the trend of average profit with the expectation construction scheme closely synchronizes with the benchmark. Conversely, the random sampling scheme encounters fluctuations that diverge from the benchmark at specific moments, resulting in marginally lower profits. Further analysis reveals that, the expectation construction scheme can be more flexible, bringing the rented capacity closer to the benchmark. The execution complexity of PAS is lower than that of the optimal, while still yielding satisfactory results.

The performance of the same options across different demand environments remains relatively consistent, with the average profit of PAS varying by about 10%. It can be observed that when demand does not experience abrupt changes, PAS exhibits good adaptability and flexible configuration, facilitating efficient operations (simple options and schemes).

#### 4.1.2 Sensitivity analysis of options.

During the experiment (group 7), we applied multiple combinations of different options to the same demand data stream to assess their impact on PAS and its robustness. For both distributions, the ^*q*^_*low*_ varies from 4 to 9.5 and ^*q*^_*high*_ from 10.5 to 16, with a step size of 0.5. This research did not prioritize extreme precision in the options, as PAS aims to provide straightforward and feasible decision-making methods. Overly detailed option settings would increase implementation difficulty.

**Fig 6:**
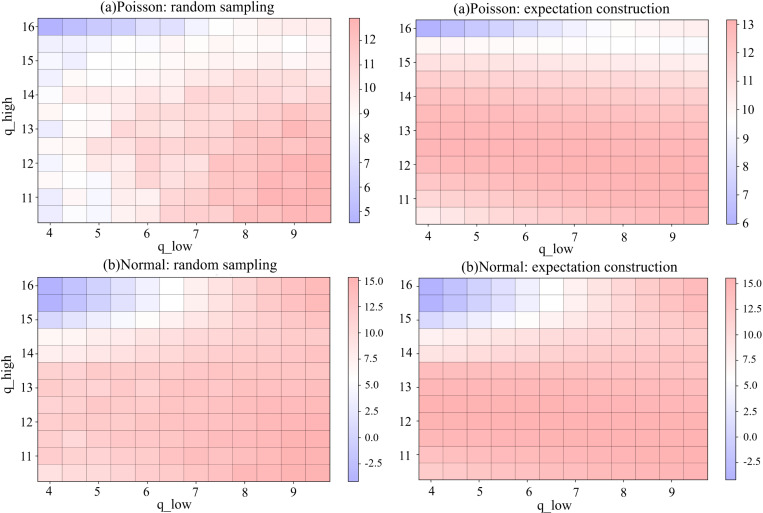
Average profits of various options under two schemes for (a) Poisson and (b) Normal distributions.

The experiments (Fig 6) show that keeping options within 1.5 to 2 times the standard deviation leads to better performance. With random sampling, the maximum gap between PAS and the benchmark approaches 30% for both distributions. Meanwhile, the expectation construction scheme shows a discrepancy of less than 20%. Values within one standard deviation yield the best profit performance. Excessively high or low options (distant from the sample mean) diminish results, with ^*q*^_*high*_ exerting a greater negative impact in the Normal distribution.

One notable characteristic of PAS is its adaptability, which allows for similar outcomes through various combinations of dual reference points and probabilities. In PAS, options are crucial; however, in information-limited environments, a simplified scheme can still deliver satisfactory results. It valuable for enterprises that cannot fully and precisely capture all data. Nevertheless, the experiments suggest that once a certain range is achieved, further refining options does not appreciably improve profits.

When using the expectation construction scheme in the Normal distribution, PAS can exceed the benchmark in average profit. With the option combination set to ^*q*^_*low*_ = 9 and ^*q*^_*high*_ = 11.5, the average profit under PAS was slightly higher than that of the benchmark (15.5506 > 1 5.55). In this experiment, the improvement is quite limited. If the options are suitably chosen, this phenomenon of surpassing may manifest in different demand streams, and the extent of the surplus increase.

### 4.2 Experiments in differentiated scenario

In this experiment, demand is assumed to follow a normal distribution, with an increased standard deviation causing greater volatility. The sample mean and standard deviation are estimated as 49.75 and 9.982, with the options set to ^*q*^_*low*_ = 40 and ^*q*^_*high*_ = 60 (*m*_*1*_ = *m*_*2*_ = 1, *n*_*1*_ = *n*_*2*_ = 2). Other parameters are provided in Table 2. According to Propositions 1 and 2, the decision is influenced by the threshold and cost (assuming fixed sales price, goodwill loss, and salvage). We will explore the impact of varying cost discount on PAS and describe its performance at different levels of ^*q*^_*TH*_.

**Table 2:** Variables and parameters.

Related to the environment and benchmarks	
Symbol	Definition
d	the value of the demand in each period
F, f	the distribution and probability density function of the demand
p, s, l	the selling price, salvage value, and goodwill loss per unit
$c_{s}$, $c_{l}$, $c_{h}$	the rental cost per unit of Type I, Type II and Type III
$q_{ST}$, $q_{DI}$	the amount of capacity rented by enterprise in two scenarios
$q_{TH}$	the threshold for utilizing Type II
**Related to the probability-based adaptive strategy (PAS)**	
**Symbol**	**Definition**
A, B, μ	upper and lower bounds and mean of demand data streams
ρ	the regret value
$q_{s}$, $q_{h}$, $q_{l}$	the capacity of Type I, Type II and Type III
$m_{1}$, $m_{2}$, $n_{1}$, $n_{2}$	the adjustment parameters for options
$q_{low}$, $q_{high}$	the options (dual reference points)
α, (1−α)	the probability of selecting options (decision variables)

#### 4.2.1 Analysis of the impact of ^*c*^_*h*_/ ^*c*^_*l*_ and ^*q*^_*TH*_ on performance.

**Fig 7:**
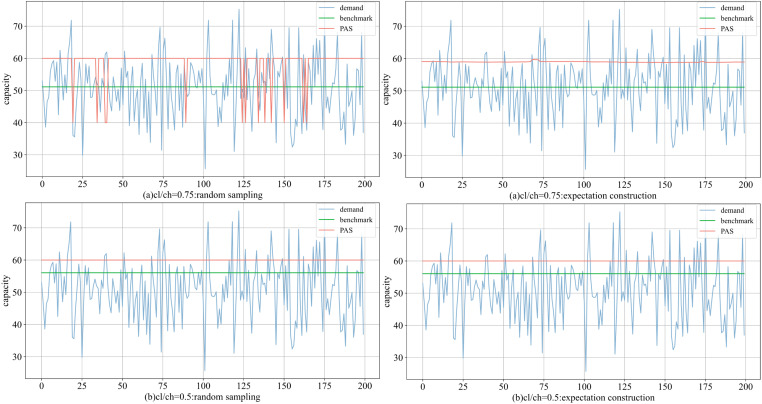
Comparison of decisions with two schemes in (a) the high-radio and (b) the low-radio.

In the high-ratio experiment (^*c*^_*h*_/ ^*c*^_*l*_ = 0.75), the parameters ^*q*^_*1*_ and ^*q*^_*2*_ (refer to Proposition 1) of the benchmark are 51.142 and 46.142, with ^*q*^_*TH*_ = 50 defined to meet the requirements for Type II. Fig 7 (group 1) illustrates that the rented capacities under both schemes exceed those of the benchmark. PAS increasingly favors ^*q*^_*high*_ when the use of Type II is permitted. In low-radio experiment (^*c*^_*h*_/ ^*c*^_*l*_ = 0.50, and ^*q*^_*1*_ = 56.046, ^*q*^_*2*_ = 46.142), the cost advantage of Type II is further highlighted, PAS stabilizes at ^*q*^_*high*_. Without sudden changes, the behavior of the two schemes will remain consistent. The discounts create a greater incentive to rent additional capacity. However, the motivation driven by cost is not absolute; It is also related to the cost ratio and the renting rules.

When the threshold is significantly higher than ^*q*^_*1*_, only Type III is used. Conversely, Type II is prioritized. This can be considered a problem within a fixed cost. Both PAS and the benchmark determine and implement similar choices. The most special condition is when the threshold is slightly higher than the ^*q*^_*1*_. The benchmark will choose to set the decision at ^*q*^_*TH*_ to reach lower costs. If ^*q*^_*high*_ is not adjusted according to the threshold, it may result in a limited rented capacity for PAS. Only high-cost equipment is usable, leading to a greater reduction in profits. The rules impose constraints on the behavior of PAS, resulting in a diminished adaptability of the strategy to demand fluctuations. For example, when ^*q*^_*TH *_= ^*q*^_*high*_ = 60, the average profit of PAS is only 20% of the optimal, or even lower; however, increasing the value of ^*q*^_*high*_ to 65–75 can raise this ratio to over 90%.

As shown in Table 5, the rise in demand volatility hinders the effectiveness of PAS, particularly when relying solely on simple combinations of options. Although the flexibility of PAS decreases, its average profit still can over 85% of the benchmark. In high-radio condition, random sampling and expectation construction reach the highest value of 0.898 and 0.939 of the benchmarks, while their lowest performances are 0.859 and 0.913, respectively. In low-ratio conditions, both schemes achieve maximum values of 0.993, while the minimum is 0.990, indicating that consistent decision-making yields the same profit outcomes.

**Table 5:** Average profits in the Normal distribution under two schemes.

	$c_{l}/c_{h}=0.75$, $q_{TH}=50$			$c_{l}/c_{h}=0.5$, $q_{TH}=50$		
	benchmark	random sampling	radio	benchmark	random sampling	radio
1	162.915	142.103	0.872	272.008	269.635	0.991
2	159.969	141.079	0.882	267.075	264.631	0.991
3	160.800	144.430	0.898	268.563	266.601	0.993
4	147.080	129.600	0.881	253.907	251.390	0.990
5	155.757	133.782	0.859	262.346	260.347	0.992
**Ave**	**157.304**	**138.199**	**0.878**	**264.780**	**262.521**	**0.991**
	benchmark	expectation construction	radio	benchmark	expectation construction	radio
**1**	162.915	153.007	0.939	272.008	269.635	0.991
**2**	159.969	147.968	0.925	267.075	264.631	0.991
**3**	160.800	149.781	0.931	268.563	266.601	0.993
**4**	147.080	134.270	0.913	253.907	251.390	0.990
**5**	155.757	143.172	0.919	262.346	260.347	0.992
**Ave**	**157.304**	**145.640**	**0.926**	**264.780**	**262.521**	**0.991**

The cost discounts provide a stronger incentive to more capacity; however, the relative size of ^*q*^_*TH*_ also restricts its selection. In complex environments, enterprises need to balance the benefits of cost discounts from increased volume against the impacts of additional capacity. When setting options, it is crucial to consider the renting rules and managerial expertise.

#### 4.2.2 Sensitivity analysis of options.

**Fig 8:**
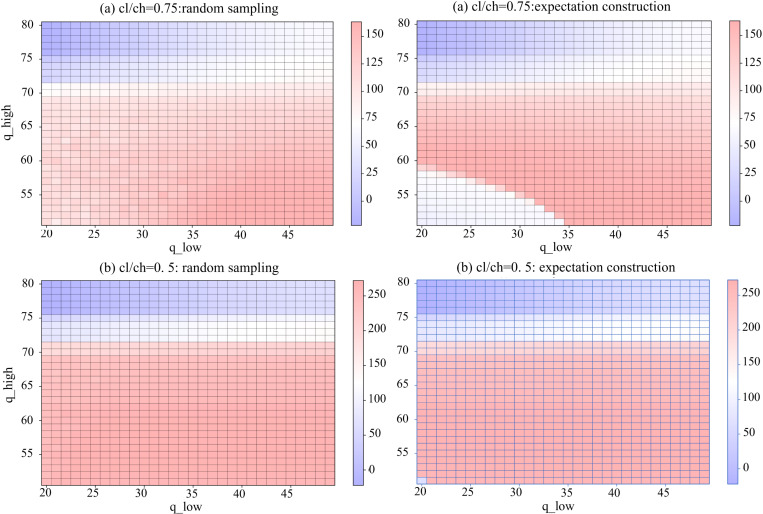
Average profits of various options under two schemes in (a) the high-radio and (b) the low-radio.

We conducted a comparative analysis of different options with a threshold set at 50. In this context, ^*q*^_*low*_ ranges from 20 to 50, while ^*q*^_*high*_ varies from 51 to 80, with a step size of 1. Fig 8 (group 1) shows that, under a high-ratio, the combinations within 1.5 times the standard deviation yield the best performance. Conversely, excessively low values of ^*q*^_*low*_ and high values of ^*q*^_*high*_ affect performance, with the latter being more significant. When the cost ratio is low, ^*q*^_*high*_ assumes a dominant role. We set ^*q*^_*high*_ > ^*q*^_*TH*_, and due to the larger cost discounts, the PAS model is more inclined to select ^*q*^_*high*_; however, if ^*q*^_*high*_ becomes excessively large and strays too far from the overall demand trend, it results in reduced profits.

Unlike experiments in 4.1, when cost differences exist, the average profit of PAS exceeds that of the benchmark in more combinations. For example, in the low-radio setting, when ^*q*^_*high*_ = 57, ^*q*^_*low*_ >= 25, both schemes achieve surpassing performance. In the high-radio setting, for the expectation construction scheme, many combinations exceed when ^*q*^_*low*_ ranges from 32 to 37 and ^*q*^_*high*_ is between 53 and 57. For the random sampling scheme, multiple options also surpass the benchmark when ^*q*^_*low*_ ranges from 38 to 49 and ^*q*^_*high*_ is 53.

## 5. Experiments in multimodal, heavy-tailed distributions and real-world dataset

In this section, we extended our experiments to consider two special types of distributions: multimodal and heavy-tailed distributions. In the standardized scenario, a multimodal distribution is constructed by combining three normal distributions with means and standard deviations of [5,10,15] and [2,3,5], respectively. Unlike normal or Poisson distributions, multimodal distributions are often asymmetric, multiple modes, and higher variability. In the differentiated scenario, a heavy-tailed distribution is generated using an exponential distribution with a scale parameter of 10. Heavy-tailed distributions are characterized by slow tail decay, lack clear boundaries, and are more prone to extreme values, making them suitable for simulating sudden events and their associated risks.

In the experiments, the environmental parameters in the standardized scenario match those in Section 4.1, while the differentiated scenario conforms to Section 4.2 with *c*_*l*_*/c*_*h*_* = 0.75* and ^*q*^_*TH*_* = 5*. We estimate sample means (11.32 and 10.24) and standard deviations (6.25 and 9.63), setting the options to (5, 17) for the multimodal and (3, 13) and (6, 12) for the heavy-tailed distribution. For the multimodal distribution, the cumulative distribution function (CDF) of the mixed distribution is calculated, and Proposition 1 is used to identify the optimal solution. For the heavy-tailed distribution, optimum is derived directly using Proposition 1.

Additionally, a real-world dataset is used to assess the performance of PAS in the differentiated scenario. The experiment utilizes publicly available data from the Pronto Cycle Share platform, focusing on rental records from the starting station BT-01 between April 2015 and October 2015. Specifically, data from April ($\hat{\mu }$=18.23, $\hat{\sigma }$=11.43) is used to define the options (^*q*^_*low*_ = 13, ^*q*^_*high*_ = 25) with ^*q*^_*TH*_* = 15*, and demand from May to October is matched.

### 5.1 Experiments in standardized scenario

#### 5.1.1 Performance of fixed options.

**Fig 9:**
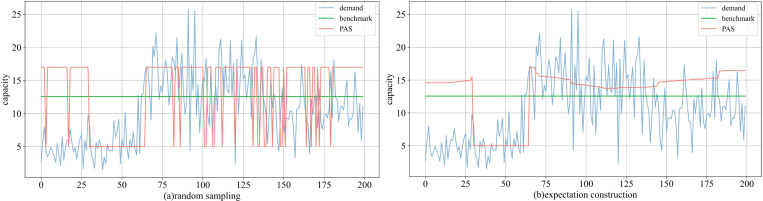
Comparison of decisions in the multimodal distribution under (a) random sampling and (b) expectation construction.

From Fig 9 (group 3), PAS exhibits the capability to perceive and adapt to demand fluctuations in multimodal distributions, irrespective of whether random sampling or expectation construction is utilized. Although the multimodal distribution results in a reduction in its fitting accuracy compared to stable distributions, PAS can capture the evolving trends by detecting variations in the magnitudes of the data. When operating within the range of a smaller peak, PAS’s selection decreases (closer to ^*q*^_*low*_), whereas within the range of a larger peak, PAS increases the rental quantity (closer to ^*q*^_*high*_).

In terms of average profit, expectation construction shows a significant advantage. Among a fixed set of options, 4 groups achieved notable profit improvements (see Table 6), with the maximum increase reaching 116.0%, while the groups that did not surpass the benchmark still achieved 90.6%. In random sampling, the best performance reached 83% of the benchmark, while the average across the 5 groups was 73.8%, with none surpassing the optimal.

**Table 6:** Average profits in the multimodal distribution under two schemes.

	benchmark	random sampling		expectation construction	
	value	value	radio	value	radio
1	8.242	5.863	0.711	8.727	1.059
2	7.505	4.959	0.661	6.797	0.906
3	8.702	6.228	0.716	9.206	1.058
4	7.623	5.901	0.774	8.843	1.160
5	7.797	6.470	0.830	8.582	1.101
**AVE**	**7.974**	**5.884**	**0.738**	**8.431**	**1.057**

Despite the increased complexity introduced by the multimodal distribution, PAS did not experience a significant decline in performance. On the contrary, in conditions where the theoretical optimal solution is more challenging to capture the variations, PAS leverages its adaptability—especially when with expectation construction—to exhibit superior matching capabilities and greater profit potential.

#### 5.1.2 Sensitivity analysis of options.

**Fig 10:**
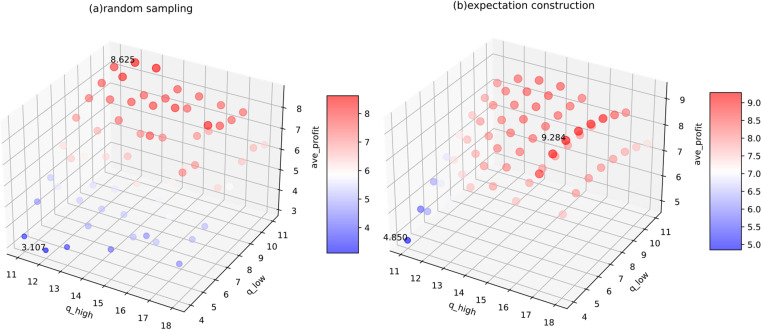
Average profits of various options in the multimodal distribution under (a) random sampling and (b) expectation construction.

We also conducted a sensitivity analysis on multiple sets of options (group 3). As shown in Section 4, confining the options within a range of $\hat{\mu }\pm \hat{\sigma }$^*q*^_*low*_ and ^*q*^_*high*_ to [4,11] and [11,18], with a step size of 1. Fig 10 shows that, under both expectation construction and random sampling, smaller values of ^*q*^_*low*_ impair profits, while larger values of ^*q*^_*high*_ have a smaller impact. When using expectation construction, we observed outperformance in 5 out of the tested combinations, with the maximum improvement reaching 106.7% (^*q*^_*low*_ = 6, ^*q*^_*high*_ = 17). However, under random sampling, no profit improvement was observed, although several combinations achieved performance close to the benchmark, exceeding 90%.

The multimodal distribution adds data complexity, but the variations show some concentration, aiding PAS in learning patterns and capturing trends. Even when perfect alignment with the data is unattainable, PAS demonstrates adaptability to demand fluctuations. For decision-makers, PAS is data-driven, and beyond selecting options, actively refining knowledge derived from data can further enhance its performance.

### 5.2 Experiments in differentiated scenario

#### 5.2.1 Analysis in heavy-tailed distribution.

In the heavy-tailed distribution, we observe phenomena that markedly differed from those in prior experiments. When designing options, they are initially defined as (5, 15). However, upon applying these to historical data, we identified a negative impact on profitability. This is related to an increase rate of tail events and a greater gap with other values (i.e., larger values raise the upper bound and mean of the data). We need to further refine the range of options, which tends to be asymmetric. Accordingly, we set (6, 12) under random sampling and (3, 13) under expectation construction.

**Fig 11:**
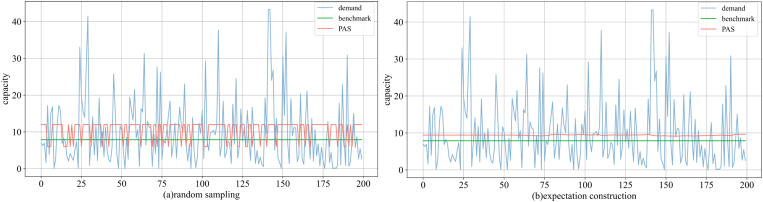
Comparison of decisions in the heavy-tailed distribution under (a) random sampling and (b) expectation construction.

As depicted in Fig 11 (group 2), in the heavy-tailed distribution, the frequency of tail events increases, and the range of data variability expands. This phenomenon diminishes the alignment between PAS and demand, making it difficult to evaluate their correspondence based on trends. Under random sampling, PAS requires more frequent adjustments. However, compared to the demand, the adjustment of PAS is lagging behind. Similarly, during expectation construction, while the changes of PAS increase, their magnitude remains small.

From Proposition 2 and Corollary 1, we know that the *α* is independent of the upper bound but is related to the mean. Therefore, larger values amplify the mean of the data; however, the differences between extreme values are not as large as expected, resulting in a small range of variation for *α* (or unchanged). Since the value of ^*q*^_*low*_ is relatively small, the fluctuations in PAS values are small.

As illustrated in the Table 7, under random sampling, the best performance reached 81.9% of the benchmark, with an average across the g groups being 73.8%. However, instances of negative profits also arise in the experiments, highlighting the challenges PAS faces in adjustment accuracy for the heavy-tailed distribution. Expectation construction showed an average ratio of 92.63%, and the highest increase reached 106.2% of the benchmark. This scheme not only outperforms random sampling, but also has the potential to surpass the benchmark.

**Table 7:** Average profits in the heavy-tailed distribution under two schemes.

	benchmark	random sampling		expectation construction	
	value	value	radio	value	radio
1	0.988	1.420	1.437	0.971	0.983
2	2.101	0.885	0.421	1.894	0.901
3	1.350	0.619	0.459	1.120	0.830
4	2.520	0.754	0.299	2.155	0.855
5	2.746	2.249	0.819	2.917	1.062
**AVE**	1.941	1.185	0.687	1.811	0.926

**Fig 12:**
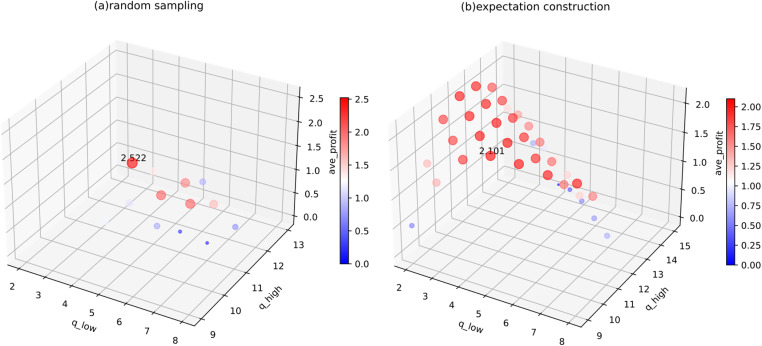
Average profits of various options in the heavy-tailed distribution under (a) random sampling and (b) expectation construction.

When the options are changed (in group 2, for random sampling, ^*q*^_*low*_ ranges from 2 to 8 and ^*q*^_*high*_ from 9 to 13; for expectation construction, ^*q*^_*low*_ ranges from 2 to 8 and ^*q*^_*high*_ from 9 to 15, with a step size of 1), we plot all positive profit outcomes (Fig 12). It is observed that the profit performance of PAS deteriorates under heavy-tailed distributions compared to other distributions, with viable combinations concentrated within $\hat{\mu }\pm$0.5$\hat{\sigma }$. Specifically, under random sampling, approximately 60% of the combinations yield negative profits, when ^*q*^_*low*_ is between 2 and 4 and ^*q*^_*high*_ equals 13. Although the combination (6, 9) produces a profit above the benchmark (2.522), its stability is poor; more commonly, profits hover around 1.5 (roughly 65%). Using expectation construction significantly improves profits, with most combinations stabilizing around 2, closely matching the benchmark. A small proportion (5%) of combinations still yield negative profits, due to high values of ^*q*^_*high*_. Although expectation construction does not surpass the benchmark in this group, it demonstrates the ability to outperform in other groups.

PAS faces challenges of instability and dispersed volatility in heavy-tailed distributions. The random sampling, which uses dual reference points, struggles to perform effectively as it cannot directly capture accurate trends from the data characteristics. Expectation construction shows better adaptability, primarily due to the regulation through linear combinations of probabilities and options. PAS’s adaptability allows decision-makers to handle stochasticity within a stable framework, despite not explicitly addressing outliers.

#### 5.2.2 Analysis in real-world dataset.

Given the unknown distribution of the real-world dataset, we employed the empirical distribution to capture its statistical properties and applied numerical methods to approximate the optimal solution. The optimal rental quantity is 23.00, resulting in an average profit of 47.30. Based on historical data of April, we chose 13 and 25 as options, close to $\hat{\mu }\pm$0.5$\hat{\sigma }$.

**Fig 13:**
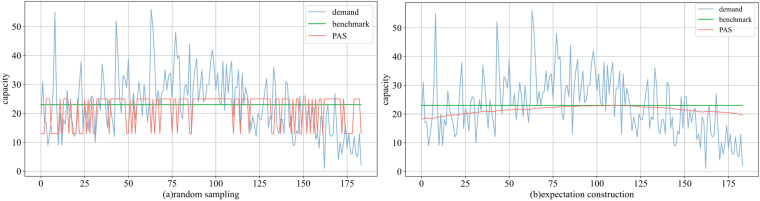
Comparison of decisions in the real-world dataset under (a) random sampling and (b) expectation construction.

As shown in Fig 13, both schemes of PAS closely track the fluctuations in demand. The overall demand pattern is characterized by a slightly higher middle range, followed by the front range, and the smallest in the rear range. Under random sampling, PAS favors ^*q*^_*high*_ in the middle range, and expectation construction results in a middle peak and lower sides. In terms of average profit, PAS achieves 37 with random sampling and 48.643 with expectation construction. The former represents 78.22% of the benchmark, while the latter surpasses by 2.84%.

**Fig 14:**
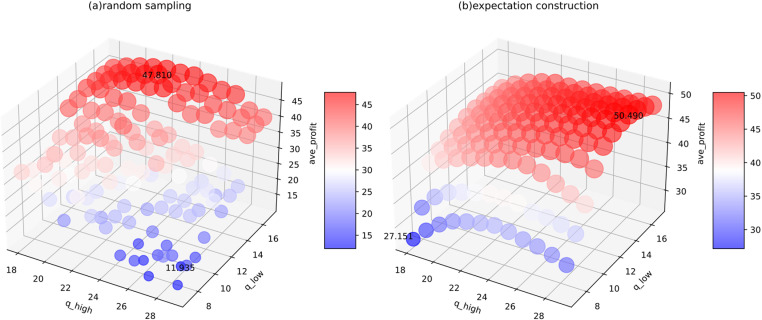
Average profits of various options in the real-world dataset under (a) random sampling and (b) expectation construction.

As the options vary (Fig 14), both a lower ^*q*^_*low*_ and a higher ^*q*^_*high*_ reduce profit. The best performance is achieved within $\hat{\mu }\pm$0.5$\hat{\sigma }$. Under random sampling, the highest value (47.810) is obtained with 16 and 23, slightly exceeding the benchmark. For expectation construction, over 35% of combinations surpassed the baseline. The maximum value (50.490) is reached with 28 and 30.

In real-world datasets, both schemes of PAS are proved effective. We find that PAS can reliably track the overall trends in data changes, generally ensuring that profits do not deteriorate. Similar to previous findings, however, PAS exhibits limited robustness in handling outliers, suggesting opportunities for further profit enhancement.

## 6. Conclusion and future research

### 6.1 Conclusion

Capacity sharing has emerged as a transformative force in shared manufacturing, driving both practical and academic domains. Shared platforms enhance efficiency by offering diverse capacity rental scenarios, enabling on-demand production usage with time- or volume-based payment. It is necessary for enterprises to adopt strategies to manage demand fluctuations and optimize production scheduling in information-constrained markets.

This paper develops a capacity sharing model that comprises suppliers, a user, and a shared platform, detailing both standardized and differentiated scenarios. We propose the probability-based adaptive strategy (PAS). This strategy uses characteristic values from partial demand data—upper and lower bounds, and the mean—along with human psychology and behavior. Options define ranges based on data and knowledge, while probabilities drive the adjustment of decisions. Unlike direct modification, PAS enables adaptive decision-making through options and probabilities. We offer two schemes to determine the final decision: random sampling and expected construction. The former emphasizes standardized and straightforward management, while the latter is weighted toward greater flexibility and efficacy.

Existing research utilizes game-theoretic approaches to establish equilibrium decisions between two enterprises, with demand represented as a function of price. When the platform is involved, it often serves as the intermediary or complement for capacity (like [33] and [34]). Our model focuses on specific rental scenarios offered by the shared platform, highlighting the adaptive decision-making of capacity users in navigating market uncertainties. While Zhao et al. [33] utilized a uniform distribution to simulate demand, we applied Poisson, Normal, multimodal, and heavy-tailed distributions, conducting experiments on real-world datasets to comprehensively validate the capabilities of PAS.

Through experimental analysis, PAS outperforms when the demand distribution is relatively stable or exhibits a concentrated trend, as Poisson, Normal, and multimodal distributions. Both random sampling and expected construction, PAS effectively detects overall changes and adapts accordingly. In differentiated scenarios, the discount rule and the threshold forces PAS to balance the cost reduction against the risk of inventory buildup, which somewhat limits its flexibility. Regarding average profit, random sampling achieves about 85% or 75% of the benchmark across three distribution types. Expected construction can exceed 95%, and even lead to higher profits.

However, in the presence of tail events and irregular fluctuations, like the heavy-tailed distribution, the performance of PAS declines. While the dual reference points ensure decision stability, they also limit PAS to handle outliers. A similar trend is observed in real-world datasets, where PAS responds to uncertainty by adjusting within a constrained range, thereby ensuring satisfactory profit levels. For enterprises, PAS provides a feasible and simple method to design options by integrating data and human refinement. Aligning options with production resources improves management efficiency. We emphasize that establishing appropriate option ranges is more critical than pursuing precision. In addition, the probability of PAS serves as a guiding factor, enabling enterprises to assess the amount of capacity to rent. However, the magnitude of probability does not indicate an absolute likelihood of selection; instead, it assists enterprises in making choices. The probability depends on environmental parameters, and timely information helps its performance. Finally, enterprises can select decision-making schemes based on their management needs or experience. Notably, close collaboration with platforms, particularly for SMEs, can reduce costs and provide additional data services.

### 6.2 Future research

This study has several limitations, which we aim to address in future research. First, a structured framework for designing options to accommodate broader scenarios needs to be developed. Second, we will explore more probability computation methods tailored to diverse environments, such as integrating risk assessment mechanisms. Third, we plan to enhance PAS’s robustness by dealing with outliers, including their identification and adjustment through collaboration of multiple approaches. Finally, the impact of differentiated costs on PAS requires further investigation, like varying cost ratios and tiered discounts.

## Supporting information

S1 Appendix Proofs of propositions. (DOCX)

S1 Data Data of experimental analysis. (XLSX)
