# Supplementary material for: Probability-based adaptive capacity rental strategy on shared platform with unknown demand distribution
Source: PLoS One. 2025 May 23;20(5):e0322837. doi: 10.1371/journal.pone.0322837 (PMC12101851; doi:10.1371/journal.pone.0322837)
Supplement: S1 Appendix — (DOCX) [file pone.0322837.s001.docx]

**S1 Appendix**

**Proof of proposition 1**

First, construct the expected profit function and let $c_{1}=c_{h},c_{2}=c_{l}$

$$\pi(q)=\left\{\begin{matrix} (p-s+l)E_{F}[\min(d,q)]-(c_{2}-s)q-lE_{F}(d),\begin{matrix} \end{matrix}q\geq q_{TH} \\ (p-s+l)E_{F}[\min(d,q)]-(c_{1}-s)q-lE_{F}(d),\begin{matrix} \end{matrix}0<q<q_{TH} \end{matrix}\right.$$

Let $q_{1}=F^{-1}(\frac{p+l-c_{2}}{p-s+l})$ and $q_{2}=F^{-1}(\frac{p+l-c_{1}}{p-s+l})$, $q_{1}>q_{2}$. The function $\pi(q)$ needs to be determined by the discontinuity point $q_{1}$, $q_{2}$ and $q_{TH}$. There are 3 scenarios that meet the conditions, which are as follows:

1. If $q_{1}\geq q_{2}\geq q_{TH}$, then $q^{*}=F^{-1}(\frac{p+l-c_{2}}{p-s+l})$
2. If $q_{1}\geq q_{TH}\geq q_{2}$, then $q^{*}=\left\{\begin{matrix} F^{-1}(\frac{p+l-c_{1}}{p-s+l}),\begin{matrix} \end{matrix}\pi(q_{2})\geq\pi(q_{1}) \\ F^{-1}(\frac{p+l-c_{2}}{p-s+l}),\begin{matrix} \end{matrix}\pi(q_{2})<\pi(q_{1}) \end{matrix}\right.$
3. If $q_{TH}\geq q_{1}\geq q_{2}$, then $q^{*}=\left\{\begin{matrix} F^{-1}(\frac{p+l-c_{1}}{p-s+l}),\begin{matrix} \end{matrix}\pi(q_{2})\geq\pi(q_{TH}) \\ q_{TH},\begin{matrix} \begin{matrix} & & & \end{matrix} \end{matrix}\pi(q_{2})<\pi(q_{TH}) \end{matrix}\right.$

Above all, we can obtain the solution.

If $q_{1}\geq q_{TH}\geq q_{2}$ and $(p+l-c_{1})q_{2}-(p+l-c_{2})q_{1}\geq(p+l-s)[\int_{0}^{q_{2}} F(x)dx-\int_{0}^{q_{1}} F(x)dx]$, or $q_{TH}\geq q_{1}\geq q_{2}$ and $(p+l-c_{1})q_{2}-(p+l-c_{2})q_{TH}\geq(p+l-s)[\int_{0}^{q_{2}} F(x)dx-\int_{0}^{q_{TH}} F(x)dx]$, then

$$q^{*}=q_{2}=F^{-1}(\frac{p+l-c_{1}}{p-s+l})$$

(ii). If $q_{1}\geq q_{TH}\geq q_{2}$ and $(p+l-c_{1})q_{2}-(p+l-c_{2})q_{1}\leq(p+l-s)[\int_{0}^{q_{2}} F(x)dx-\int_{0}^{q_{1}} F(x)dx]$, or$q_{1}\geq q_{2}\geq q_{TH}$, then

$$q^{*}=q_{1}=F^{-1}(\frac{p+l-c_{2}}{p-s+l})$$

(iii). If $q_{TH}\geq q_{1}\geq q_{2}$ and $(p+l-c_{1})q_{2}-(p+l-c_{2})q_{1}\leq(p+l-s)[\int_{0}^{q_{2}} F(x)dx-\int_{0}^{q_{TH}} F(x)dx]$, then

$$q^{*}=q_{TH}$$

**Proof of proposition 2**

First, construct the expected profit function and let $q_{1}=q_{low},q_{2}=q_{high}$, $c_{1}=c_{h},c_{2}=c_{l}$.

$$\pi_{DI}(q_{1},q_{2}|\alpha)=\left\{\begin{matrix} (p-s+l)E_{F}[\min(d,q_{1})]-(c_{h}-s)q_{1}-lE_{F}(d)=\pi_{1}(q_{1}),\begin{matrix} \end{matrix}\alpha\\ (p-s+l)E_{F}[\min(d,q_{2})]-(c_{l}-s)q_{2}-lE_{F}(d)=\pi_{2}(q_{1}),\begin{matrix} \end{matrix}1-\alpha\end{matrix}\right.$$

$$E[\pi(q_{1},q_{2}|\alpha)]=\alpha E[\pi_{1}(q_{1})]+(1-\alpha)E[\pi_{2}(q_{2})]$$

$$\alpha E[\pi_{1}(q_{1})]=\alpha\{(p-s+l)\int_{0}^{\infty} \min(q_{1},x)dF(x)+sq_{1}-lE[d]-c_{1}q_{1}\}$$

$$(1-\alpha)E[\pi_{2}(q_{2})]=(1-\alpha)\left\{(p-s+l)\int_{0}^{\infty} \min(q_{2},x)dF(x)+sq_{2}-lE[d]-c_{2}q_{2}\right\}$$

Construct the objective function for the minimax regret criterion.

(1.1)

Problem (1.1) can be transformed as the following.

Next, solve the problem layer by layer, starting with the innermost layer .

(1.2)

We solve problem (1.2). By observing the form of the function, we can see that finding the maximum value of this expression is equivalent to finding the maximum value of its inner function. Let $\Omega$ be the known support set of the demand distribution, and $M_{i}$be the known partial information. Establish the following objective function:

(1.3)

Following the idea of robust optimization, perform a dual transformation on the original problem.

$\begin{matrix} \min_{a_{0},...,a_{n}} \begin{matrix} & \sum_{i=0}^{n} a_{i}M_{i} \end{matrix} \\ s.t.\begin{matrix} & \sum_{i=0}^{n} a_{i}x^{i}-(\beta-\alpha) \end{matrix}(p-s+l)[\min(q_{1},x)-\min(q_{2},x)]\in\xi* \end{matrix}$ (1.4)

Then, solve problem (1.4). According to the Proposition 1(a)(b) from Perakis and Roels, classify and discuss the problem.

1. The function is concave on the interval $\beta\in[0,\alpha]$and on the semi-interval $\beta\in[\alpha,1]$, but not necessarily on $[0,1]$.
2. The function $\rho(\alpha)$ is convex. Moreover, $\alpha^{\text{*}}=\mathrm{argmin}\rho(\alpha)$is such that:

$$\max_{\beta\in[0,\alpha^{*}]}G(\beta;\alpha^{*})=\max_{\beta\in[\alpha^{*},1]}G(\beta;\alpha^{*})$$

Problem (1.4) needs to satisfy $\sum_{i=0}^{n} a_{i}x^{i}=(\beta-\alpha)(p-s+l)[\min(q_{1},x)-\min(q_{2},x)]$, which means solving for the intersection points between these two functions.

Let$\begin{matrix} h(x)=(\beta-\alpha)(p-s+l)[\min(q_{1},x)-\min(q_{2},x)] \\ =\left\{\begin{matrix} 0,\begin{matrix} & x\leq q_{1} \end{matrix} \\ (\beta-\alpha)(p-s+l)(q_{1}-x),\begin{matrix} & q_{1}<x<q_{2} \end{matrix} \\ (\beta-\alpha)(p-s+l)(q_{1}-q_{2}),\begin{matrix} & x\geq q_{2} \end{matrix} \end{matrix}\right. \end{matrix}$.

If $\beta>\alpha$, one case satisfies the condition, namely $\alpha_{0}=\alpha_{1}=0$. The maximum regret is equal to $\begin{matrix} \max_{\beta\in[\alpha,1]}(\beta-\alpha)(s-c_{1})q_{1}-(\beta-\alpha)(s-c_{2})q_{2} \\ =\left\{\begin{matrix} (1-\alpha)(s-c_{1})q_{1}-(1-\alpha)(s-c_{2})q_{1},\begin{matrix} \end{matrix}\frac{c_{2}-s}{c_{1}-s}\geq\frac{q_{1}}{q_{2}} \\ \begin{matrix} & & & \end{matrix}0,\begin{matrix} \begin{matrix} \end{matrix} & & \end{matrix}\begin{matrix} & & & \frac{c_{2}-s}{c_{1}-s}\geq\frac{q_{1}}{q_{2}} \end{matrix} \end{matrix}\right. \end{matrix}$. When $\alpha_{0}=\alpha_{1}\neq0$, the range of values for the mean $\mu$ contradicts our model's assumptions; therefore, this case does not exist.

If $\beta\leq\alpha$, one cases satisfy the condition. When the functions have two intersection points, the coordinates are $(A,0)$ and $(q_{2},(\beta-\alpha)(p-s+l)(q_{1},q_{2}))$. Based on the intersection point, we get $\alpha_{0}=-A\frac{(\beta-\alpha)(p-s+l)(q_{1}-q_{2})}{(q_{2}-A)}$ and $\alpha_{1}=\frac{(\beta-\alpha)(p-s+l)(q_{1}-q_{2})}{(q_{2}-A)}$. At this time, $\alpha_{0}+\mu\alpha_{1}=\frac{(\mu-A)(\beta-\alpha)(p-s+l)(q_{1}-q_{2})}{(q_{2}-A)}$. When $\alpha_{0}=\alpha_{1}=0$, the range of values for the mean $\mu$ contradicts our model's assumptions; therefore, this case does not exist. Overall, the maximum regret is given by the following expression:

$$\begin{matrix} \max_{\beta\in[0,\alpha]}\left(\frac{(\mu-A)(\beta-\alpha)(p-s+l)(q_{1}-q_{2})}{q_{2}-A}+(\beta-\alpha)(s-c_{1})q_{1}-(\beta-\alpha)(s-c_{2})q_{2}\right) \\ =(\beta-\alpha)\left[\frac{(\mu-A)(p-s+l)(q_{1}-q_{2})}{q_{2}-A}+(s-c_{1})q_{1}-(s-c_{2})q_{2}\right] \end{matrix}$$

If $\frac{(\mu-A)(p-s+l)(q_{1}-q_{2})}{q_{2}-A}>(s-c_{2})q_{2}-(s-c_{1})q_{1}$, the above expression equals 0.

Conversely, the above expression equals $-\alpha\left[\frac{(\mu-A)(p-s+l)(q_{1}-q_{2})}{q_{2}-A}+(s-c_{1})q_{1}-(s-c_{2})q_{2}\right]$.

Last, let $\max_{\beta\in[0,\alpha^{*}]}G(\beta;\alpha^{*})=\max_{\beta\in[\alpha^{*},1]}G(\beta;\alpha^{*})$.

$\left\{\begin{matrix} (1-\alpha)(s-c_{1})q_{1}-(1-\alpha)(s-c_{2})q_{2}=0, & \text{ }(c_{2}-s)q_{2}-(c_{1}-s)q_{1}\geq\mu\frac{(p-s+l)(q_{2}-q_{1})}{q_{2}} \\ (1-\alpha)(s-c_{1})q_{1}-(1-\alpha)(s-c_{2})q_{2}=B(\alpha), & 0\leq(c_{2}-s)q_{2}-(c_{1}-s)q_{1}\leq\mu\frac{(p-s+l)(q_{2}-q_{1})}{q_{2}} \\ 0=B(\alpha), & \text{ }(c_{2}-s)q_{2}-(c_{1}-s)q_{1}\leq0 \end{matrix}\right.$, with $B(\alpha)=-\alpha\left[\frac{(\mu-A)(p-s+l)(q_{1}-q_{2})}{q_{2}-A}+(s-c_{1})q_{1}-(s-c_{2})q_{2}\right]$

$$\alpha^{*}=\left\{\begin{matrix} 1, & \text{ }(c_{2}-s)q_{2}-(c_{1}-s)q_{1}\geq\frac{(\mu-A)(p-s+l)(q_{2}-q_{1})}{q_{2}-A} \\ \frac{(c_{1}-s)q_{1}-(c_{2}-s)q_{2}}{(\mu-A)(p-s+l)(q_{1}-q_{2})}\cdot(q_{2}-A), & \text{ }0\leq(c_{2}-s)q_{2}-(c_{1}-s)q_{1}\leq\frac{(\mu-A)(p-s+l)(q_{2}-q_{1})}{q_{2}-A} \\ 0, & \text{ }(c_{2}-s)q_{2}-(c_{1}-s)q_{1}\leq0 \end{matrix}\right.$$

**Proof of corollary 1**

When $0\leq(c_{2}-s)q_{2}-(c_{1}-s)q_{1}\leq\frac{(\mu-A)(p-s+l)(q_{2}-q_{1})}{q_{2}-A}$，$\alpha^{*}=\frac{(q_{2}-A)[(c_{1}-s)q_{1}-(c_{2}-s)q_{2}]}{(\mu-A)(p-s+l)(q_{1}-q_{2})}$.

Let $N=(q_{2}-A)[(c_{1}-s)q_{1}-(c_{2}-s)q_{2}]$, $D=(\mu-A)(p-s+l)(q_{1}-q_{2})$, we will derive the expression with respect to each parameter.

Derive from $A$: $\left(\frac{N}{D}\right)^{'}_{A}=\frac{[(c_{1}-s)q_{1}-(c_{2}-s)q_{2}](q_{2}-\mu)}{(\mu-A)^{2}(p-s+l)(q_{l}-q_{2})}\geq0$

Derive from $\mu$: $\left(\frac{N}{D}\right)^{'}_{\mu}=\frac{-\left[(c_{1}-s)q_{1}-(c_{2}-s)q_{2}\right](q_{2}-A)}{(\mu-A)^{2}(p-s+l)(q_{1}-q_{2})}\leq0$

Derive from $p$: $\left(\frac{N}{D}\right)^{'}_{p}=-\frac{[(c_{1}-s)q_{1}-(c_{2}-s)q_{2}](q_{2}-A)}{(\mu-A)(p-s+l)^{2}(q_{1}-q_{2})}\leq0$

Derive from $l$: $\left(\frac{N}{D}\right)^{'}_{l}=-\frac{[(c_{1}-s)q_{1}-(c_{2}-s)q_{2}](q_{2}-A)}{(\mu-A)(p-s+l)^{2}(q_{1}-q_{2})}\leq0$

Derive from $s$: $\left(\frac{N}{D}\right)^{'}_{s}=\frac{(q_{2}-A)\left[(q_{2}-q_{1})(p+l)+c_{1}q_{1}-c_{2}q_{2}\right]}{(\mu-A)(p-s+l)^{2}(q_{1}-q_{2})}$, if $\frac{q_{2}}{q_{1}}<\frac{p+l-c_{1}}{p+l-c_{2}}$, $\left(\frac{N}{D}\right)^{'}_{s}>0$, else $\left(\frac{N}{D}\right)^{'}_{s}\leq0$.

Derive from $c_{1}=c_{high}$: $\left(\frac{N}{D}\right)^{'}_{c{}_{1}}=\frac{q_{1}(q_{2}-A)}{(\mu-A)(p-s+l)(q_{1}-q_{2})}\leq0$

Derive from $c_{2}=c_{low}$: $\left(\frac{N}{D}\right)^{'}_{c_{2}}=\frac{-q_{2}\cdot(q_{2}-A)}{(\mu-A)\cdot(p-s+l)\cdot(q_{1}-q_{2})}\geq0$

Derive from $q_{1}=q_{low}$: $\left(\frac{N}{D}\right)^{'}_{q_{1}}=\frac{(q_{2}-A)(c_{2}-c_{1})q_{2}}{(\mu-A)(p-s+l)(q_{1}-q_{2})^{2}}\leq0$

Derive from $q_{2}=q_{high}$:

$$\left(\frac{N}{D}\right)^{'}_{q_{2}}=\frac{(c_{1}-s)q_{1}(q_{1}-A)-(c_{2}-s)q_{2}(q_{1}-A)-(c_{2}-s)(q_{2}-A)(q_{1}-q_{2})}{(\mu-A)(p-s+l)(q_{1}-q_{2})^{2}}$$

If $(c_{1}-s)q_{1}-(c_{2}-s)q_{2}\geq\frac{(c_{2}-s)(q_{2}-A)(q_{1}-q_{2})}{q_{1}-A}$, $\left(\frac{N}{D}\right)^{'}_{q_{2}}\geq0$, else $\left(\frac{N}{D}\right)^{'}_{q_{2}}<0$.

**Proof of corollary 2**

Let $c_{1}=c_{2}=c$, the $\alpha^{*}$ in Proposition 2 satisfies:

$$\alpha^{*}=\left\{\begin{matrix} 1,\begin{matrix} & & \begin{matrix} & & & \end{matrix} & c-s\geq\frac{(\mu-A)(p-s+l)}{q_{2}-A} \end{matrix} \\ \frac{(c-s)(q_{2}-A)}{(\mu-A)(p-s+l)},\begin{matrix} \end{matrix}0\leq c-s\leq\frac{(\mu-A)(p-s+l)}{q_{2}-A} \end{matrix}\right.$$
